# Online Temptations: COVID-19 and Religious Misinformation in the MENA Region

**DOI:** 10.1177/2056305120948251

**Published:** 2020-07-30

**Authors:** Mahsa Alimardani, Mona Elswah

**Affiliations:** University of Oxford, UK

**Keywords:** religious misinformation, the MENA region, digital religion

## Abstract

During the coronavirus pandemic, religious misinformation has been found on social media platforms causing fear, confusion, and polluting the Middle East and North Africa (MENA) region’s online sphere. Exploring cases of religious clickbait in the form of false hadiths and viral religious advice from religious figures entrenched in the MENA’s political elite, this essay discusses how new dynamics for religion in the age of the Internet are contributing to a uniquely regional and religious form of misinformation. This essay looks at how the phenomenon of religious misinformation is a defining characteristic of the MENA’s online sphere, becoming even more acute during the COVID-19 pandemic.

## Introduction

COVID-19’s impact on online communications assumes multiple shapes and forms. Within the Middle East and North Africa (MENA) region, long-standing issues of communication contribute to the “infodemic” that has been emerging alongside this pandemic. Aggressive censorship regimes and a deficit of public trust in governments and authorities make the pursuit of credible sources for news and information difficult. Characteristic within the MENA region’s infodemic is the phenomenon of *religious misinformation* persisting through social media. While not unique to the COVID-19 pandemic, its impact has become much more acute during this period. It underlines a characteristic of the region’s information space that deserves further examination.

Over the last few years, researchers have scrambled to examine different types of misinformation in relation to politics, health, and science. However, very little has been explored in terms of the phenomenon of *religious misinformation* and its unique hold within the region’s online public sphere. Religious misinformation presents misleading guidance, misinterpreting scriptures or religious records, or the false claim of divine power or knowledge. Religious misinformation draws on fear, emotional appeals, or the credibility of religious authority to persuade the recipient of these various messages. Unlike its other forms, religious misinformation is harder to fact-check and requires a deeper knowledge of religion and its sociopolitical context to discern.

The use of *religious* misinformation draws upon a powerful appeal on the role of Islam, which is deeply entrenched in the culture, society, and politics of the region. Within the MENA region, Islam has long been studied as a unifying or divisive force in Muslim-majority countries. It has been mostly studied from a political point of view to scrutinize its role in political movements in the region (Alimardani & Milan, 2018; Munson, 1988). However, what has not been explored are the possible ways of misusing Islam to create, promote, and amplify online misinformation. What we argue is that COVID-19 has underlined a unique condition for online communication in the region: the dangers and appeals of religious misinformation.

The spread of COVID-19 religious misinformation has not been unique to the MENA region. Across the world, actors promote religious remedies and give the false hope of divine immunity to “true” believers attending centers of worship. We saw examples of figures using their religious authority to encourage people to drink cow urine in India, visit churches in the United States on Easter, and attend Holy Communion, drinking from one spoon at the Greek Orthodox church. These religious directions were based on the notions that believers would be protected from contagion through their belief and sacred actions. In the MENA region, these religious appeals most often misused the appeal of Islam to promote dangerous remedies, theories, and behaviors for various intentions.

While Islam prohibits the spread of unverified information and rumors,^1^ Islamic-tailored religious misinformation has contributed to the region’s infodemic. Actors promoting or creating religious misinformation in the MENA region find themselves with powerful tools and conditions to go viral online and ultimately further endanger public health. We certainly cannot state who is behind all COVID-19 religious misinformation in the MENA region, but we can emphasize that they are produced by various types of actors in varying forms. The perpetrators of some of these messages are sometimes religious authorities with ties to ruling establishments, motivated by the aim to attract more followers or relevance during moments like a pandemic through social media. In other instances, these manipulations are promoted by content creators who fabricate, misinterpret, and misuse religious sculptures to attract followers for monetary gain.

### Fake Religious Remedies

One of the problems of religious misinformation is authority, reflected intensely within Iran. Here, clerics occupy a central position in the approval of legislation guaranteed by the Constitution of the Islamic Republic based on the Revolution’s interpretation of *velayat-e faqih* (translated as, the guardianship of the jurists). As a result, Shiite clergy sit at the center of decision-making in Iran. Preaching and advice on medical science, for example, have never remained out of bounds for this class of Iran’s religious-political elite.

These complex authority structures compounded problems of public trust. Within Iran, the Ministry of Health actively dismissed and diminished the threat of COVID-19 as the initial cases were found (BBC Persian, 2020). This tarnished the credibility of the good advice and work the Ministry provided after the early denials. However, the statements of some clerics have further entangled notions of trust and authoritative advice on the virus. For example, a member of Iran’s Assembly of Experts,^2^ Ayatollah^3^ Mohammad Mehdi Mirbaqeri, has been involved in viral social media interviews questioning the need to lockdown or socially distance when such measures were not necessary for previous illnesses or epidemics (Fars News Agency, 2020). Such advice places the authority of science and medicine in direct competition with the authority of Iran’s religious elite.

Similarly, underlining the divide that exists between Iran’s medical authorities and clerical authorities is the fringe cleric, Ayatollah Tabrizian. Tabrizian has acquired a cult following as “the father of Islamic medicine”—a practice of medicine that rejects global medical science as a pillar of western infiltration (Aramesh, 2018). As the COVID-19 crisis unfolded in Iran, Tabrizian advised followers infected with the virus to swab their anuses with a piece of cotton dipped in violet oil, according to the dictates of his school of “Islamic medicine.” His remedy went viral on his Telegram channel of over 200,000 followers and beyond onto other Persian language social media, received as ridicule and prescription. Tabrizian’s example has helped amplify Islamophobia.

While some Iranian authorities denounce Tabrizian’s opinions against medical science as “unIslamic” (Iranian Students’ News Agency, 2020), he is largely allowed to maintain his platform and clerical title. Tabrizian and other “Islamic Medicine” proponents seem to be increasing their base of supporters in Iran by taking advantage of sentiments of distrust toward the government and health system that have been mired in alleged corruption alongside the utility of social media. They are safeguarded by support they have within religious and governmental authorities (Aramesh, 2018). On the platforms Tabrizian thrives on—Telegram and Aparat—there are no regulations on his religious misinformation. On Aparat—the Iranian alternative to YouTube—content moderation rules strictly adhere to Iran’s censorship regulations (Center for Human Rights in Iran, 2017), leaving the official pages for Tabrizian to prescribe and promote remedies of “Islamic Medicine” unchecked. While the infodemic has elicited responses from many mainstream platforms to remove dangerous COVID-19 information, Telegram has done very little in the way of content removals for dangerous COVID-19 misinformation. Thus far, they have just offered a new feature of channel verification, without attempts to remove problematic content.

While we cannot say that the opposition of some clerics to medical advice has been a determining factor in the high infection and death rates in Iran, it has contributed to polarization in responses within an already fractured public struggling to find the right sources and authorities to follow for advice.

### Fake Scriptures Go Viral

Islamic scholars have long dealt with verification within scriptures. Verification of sources is important to preserve the authenticity of the sayings of the Prophet Mohammad. While the Quran and much Islamic scholarship are dedicated to information literacy, a problem of misinformation has riddled Islam for centuries (Ahmed, 2018). Among these problems is the spread of “False Hadiths”—fabrications of retellings of the Prophet’s words and deeds. Because of this problem, Hadith scholars have developed rigid methods to ascertain authenticity.

A flood of Arabic-speaking YouTube videos prophesized that a divine sound would be heard on the night of the 15th of Ramadan 2020 based on a false Hadith. As the prophecy foretold, the sound would take 70,000 souls and leave 70,000 deaf. The prophecy built its claim on the basis that the rise of COVID-19 is a sign that this is the year in which the sound will be heard. These videos relied on fear to attract Arab YouTube users, who are encouraged by the video creators to share these videos. These videos were viewed millions of times and found their way to other platforms including WhatsApp. The virality and the fear induced by the videos among millions through Arabic-online spheres led the official Egyptian religious entity *Al Azhar* to release an official statement denouncing the videos as false (Mustafa, 2020).

To this date, these clickbait videos are available on YouTube channels and are still able to attract people and earn money. They were able to escape the platforms’ algorithms, and they have not been removed by content moderators. These videos, as like most forms of religious misinformation, seem community-friendly, and to catch their falsehood, a moderator needs to be trained to understand the science of Hadith and the political economy of religious misinformation. These videos—and the majority of religious misinformation—are more likely to live longer on online platforms compared with other forms of misinformation that could be easily debunked.

## Conclusion

Issues of verification in the age of mass media, and now social media, have been a long-discussed topic among Islamic media scholars. While Islam might be a common thread in COVID-19 religious misinformation, there is nothing inherently Islamic to the religious tinged elements of misinformation on social media, beyond its use for financial or political expediency. Furthermore, religious misinformation can be used by opponents of Islam to further undermine the religion and its adherents, prompting Islamophobia.

Religious misinformation comes from various types of actors. On one hand, we saw examples of top-down misinformation from certain religious leaders who benefit from social media platforms to spread false remedies. Incidents of bottom-up misinformation, on the other hand, demonstrate content creators taking advantage of pandemic-induced uncertainty to attract new subscribers and followers. While the content and the actors behind religious misinformation are significant, in McLuhan’s terms, the medium is the real message (McLuhan, 1964). Social media platforms are defining new parameters for religious dynamics and authority. They are the impetus behind why religious misinformation is contributing to this infodemic.

Social media platforms have become digital worship spaces for some believers. In recent years, religious leaders were able to share their teachings, while repurposing and remixing Holy scripture to bolster religious participation (Brubaker & Haigh, 2017; Cheong, 2014). Social media have in some instances disrupted and challenged the traditional forms of religious authority structures. Now, anyone can claim religious authority, or assume religious leadership, something ordinarily be out of reach without social media. This form of misinformation finds a home among an online audience eager for peace at a time of crisis. Conspiracy-based content reduces the complexity of reality and simplifies causation in times of uncertainty (Vicario et al., 2016).

While mainstream platforms have often fallen short in curbing misinformation in their native English-speaking countries, their responses in non-English speaking countries may have fared worse. MENA-related content moderation issues predate the pandemic and have often been excused as a lack of local linguistic capacity (Elswah & Howard, 2020). Religious COVID-19 misinformation has further underlined the need for these companies to allocate more resources to the region and to train their content moderators to all sorts of misinformation.

Currently, researchers have allocated their time and energy to study the spread of political and health misinformation around COVID-19 on social media; however, religious misinformation remains largely unexamined. Unfamiliarity with the mechanisms of verifying religious scriptures; language limitations; misunderstanding the impact of this misinformation; and a sense of hesitancy and sensitivity when criticizing issues related to Islam have been some of the hurdles. While this article has mainly focused on religious misinformation, there are positive uses of religious authority, Hadith and Quran verses that have been circulating on social media during the pandemic to encourage people to avoid harming others, maintain hygiene, and employ social distancing measures. We have only presented a snapshot of the relevance of religious misinformation during COVID-19 from an Islamic point of view. We encourage further research on this phenomenon in its relation to other religions and sociopolitical contexts.

## Notes

1. “Do not follow that of which you have no knowledge. Surely the hearing, the sight, the heart—each of these shall be called to account” (The Quran, 17:36). “O you who have believed, if there comes to you a disobedient one with information, investigate, lest you harm a people out of ignorance and become, over what you have done, regretful.” (The Quran, 49:6).

2. The Assembly of Experts is a body of high-ranking Iranian clerics tasked with selecting the Supreme Leader and keeping a check on him and his office.

3. The title of the Ayatollah is an honorific given to Shiite Islamic clerics by the Shiite seminaries of the city of Qom, after passing certain levels of education within that system.
